# ERα Signaling Increased IL-17A Production in Th17 Cells by Upregulating IL-23R Expression, Mitochondrial Respiration, and Proliferation

**DOI:** 10.3389/fimmu.2019.02740

**Published:** 2019-11-27

**Authors:** Hubaida Fuseini, Jacqueline-Yvonne Cephus, Pingsheng Wu, J. Brooke Davis, Diana C. Contreras, Vivek D. Gandhi, Jeffrey C. Rathmell, Dawn C. Newcomb

**Affiliations:** ^1^Department Pathology, Microbiology and Immunology, Vanderbilt University, Medical Center North, Nashville, TN, United States; ^2^Department of Medicine, Vanderbilt University Medical Center, Medical Center North, Nashville, TN, United States

**Keywords:** Th17, estrogen receptor alpha, IL-23R, IL-17A, cytochrome c oxidase

## Abstract

Women have increased prevalence of Th17-mediated autoimmune diseases, including lupus and multiple sclerosis, and severe asthma. While estradiol and progesterone increased IL-17A production in Th17 cells by inhibiting *Let7f* miRNA expression and increasing IL-23 receptor (IL-23R) expression, it remained unclear how estrogen signaling through the canonical nuclear receptors, estrogen receptor α (ERα) and/or ERβ, regulated this pathway. We hypothesized that estrogen signaling through ERα increased IL-23R expression and IL-17A production from Th17 cells. To test this hypothesis, naïve T cells from WT female, WT male, *Esr1*^−/−^ and *Esr2*^−/−^ female mice were differentiated into Th17 cells. IL-17A production and IL-23R expression were significantly increased in Th17 cells from WT female mice compared to Th17 cells from WT male mice. Deletion of ERα (*Esr1*^−/−^), but not ERβ (*Esr2*^−/−^), significantly decreased IL-17A production and IL-23R expression in Th17 cells by limiting IL-23R expression in a *Let-7f* dependent manner. ERα deficiency also decreased Th17 cell proliferation as well as decreased T cell metabolism as measured by ATP-linked oxygen consumption rate and proton leakage. Further, we found that *Cox20* expression, a protein involved in mitochondrial respiration through assembly of cytochrome c oxidase in the electron transport chain, was increased in Th17 cells from WT female mice compared to Th17 cells from WT male and *Esr1*^−/−^ female mice. Inhibition of Cox20 decreased IL-17 production in Th17 cells from WT female mice. Combined these studies showed that ERα signaling increased IL-17A production in Th17 cells by upregulating IL-23R expression and promoting mitochondrial respiration and proliferation.

## Introduction

A sexual dimorphism exists in immune responses. As adults, women have an increased prevalence of autoimmune diseases, including lupus and multiple sclerosis, as well as asthma and allergic diseases compared to men (1–5). In females, asthma prevalence increases around the time of puberty, and 30–40% of women report cyclic changes in asthma control, with decreased lung function and increased symptoms during the pre and peri-menstrual phase of the menstrual cycle (5–9). Further, during pregnancy approximately two-thirds of women with asthma reported either increased or decreased asthma symptoms compared to asthma symptoms prior to pregnancy, with increased asthma symptoms more prominent in patients with severe phenotypes of asthma (10–12). Similarly, in lupus and multiple sclerosis, disease prevalence is increased after puberty and symptoms improve during pregnancy (13–15). These findings suggest that ovarian hormones are important in pathogenesis of asthma, allergic diseases, lupus, and multiple sclerosis.

CD4+ Th17 cells are increased in patients with lupus, multiple sclerosis, and severe phenotypes of asthma (16, 17). Th17 cells produce interleukin 17A (IL-17A) leading to increased neutrophil infiltration and inflammation (18, 19). Th17 cell differentiation requires T cell activation in the presence of IL-6, IL-1β (for human cells), and low concentrations of TGF-β (20–22), leading to increased expression of *Rorc* (RORγT) expression and IL-17A production (18, 23). IL-23 is not required for Th17 cell differentiation. However, IL-23 signaling through the IL-23 receptor (IL-23R) increases IL-17A production and is important in pathogenesis of autoimmune diseases and potentially asthma (17, 24).

T cell metabolism is also important for T cell differentiation after activation. Th1, Th2, and Th17 cells rely on glycolysis to meet metabolic needs for differentiation (25). Th17 cells were recently shown to require glutaminolysis and utilize oxidative phosphorylation and *de novo* fatty acid synthesis for IL-17A production (26–30). With the known sex bias in Th17 diseases, sex hormones may also alter T cell metabolism and Th17 cell differentiation.

Our previous findings showed that ovarian hormones, including estrogen and progesterone are important in Th17 cell differentiation. Estrogen and progesterone increased IL-23R expression and IL-17A production from Th17 cells as well as increased IL-17A-mediated airway inflammation (24). *Let-7f* microRNA inhibited IL-23R expression on Th17 cells (31), and our findings further showed that estrogen and progesterone inhibited *Let-7f* microRNA expression, leading to increased IL-23R expression and increased IL-17A protein expression in Th17 cells (24). Therefore, these data showed a mechanism by which estrogen and progesterone increased IL-17A protein expression in Th17 cells.

Estrogen most commonly signals by binding to the nuclear hormone receptors, estrogen receptor α (ERα) and β (ERβ). Once bound, the estrogen-ER complex regulates transcription of target genes by binding directly to estrogen response elements on DNA or indirectly binding through protein-protein interactions with transcription factors (32, 33). ERα and ERβ are expressed in CD4+ T cells, and ER signaling enhances IFN-γ production from Th1 cells and has variable effects on IL-4 production from Th2 cells and IL-17A production from Th17 cells (33).

In a mouse model of colitis, selective ERα deficiency in CD4+ T cells inhibited IL-17A and IFNγ production from Th17 and Th1 cells, respectively, in the mesenteric lymph nodes as well as decreased Th17 and Th1-mediated inflammation in the gut (34). However, in an experimental autoimmune encephalomyelitis (EAE) mouse model of multiple sclerosis, estrogen signaling through ERα or ERβ decreased Th17 and/or Th1 induced EAE inflammation (35, 36). ERα signaling also increased mitochondrial respiration while ERα deletion in CD4+ T cells decreased the oxygen consumption rate (OCR) and ATP production (34, 37). However, it remained unclear how estrogen signaling through ERα or ERβ altered Th17 cell metabolism and IL-17A production. We hypothesized that estrogen signaling through ERα increased IL-23R expression and IL-17A production from Th17 cells. Our findings showed that ERα deficiency downregulated IL-23R expression, mitochondrial respiration, and proliferation on Th17 cells leading to decreased IL-17A production.

## Materials and Methods

### Mice

WT female, WT male, ERα female knockout (*Esr1*^−/−^, stock number 004744) and ERβ female knockout (*Esr2*^−/−^, stock number 004745) C57BL/6J mice were purchased from Jackson Laboratory (Bar Harbor, ME). Breeding colonies were established at Vanderbilt University Medical Center, and littermate WT controls were used for all experiments with *Esr1*^−/−^ or *Esr2*^−/−^ female mice. All animal experiments were conducted in adherence to the rules and regulations of the Association for Assessment and Accreditation of Laboratory Animal Care and were approved by the International Animal Care and Use Committee at Vanderbilt University Medical Center.

### Isolation and FACS Sorting of Naïve CD4+ T Cells

CD4+ T cells were enriched from the spleens of mice using a commercially available murine CD4+ T cell enrichment kit (Miltenyi). Naive CD4+ T cells, defined as CD3+CD4+CD62L+ T cells, were FACS sorted using APC conjugated anti-mouse CD3 antibody (clone 145-2C11), FITC conjugated anti-mouse CD4 antibody (clone H129.19), and PE conjugated anti-mouse CD62L antibody (clone MEL-14). Post-sort analysis showed >96% purity of naïve T cells.

### Th1, Th2, and Th17 Cell Differentiation From Murine Naïve CD4 T Cells

Naïve CD4+ T cells were activated with anti-CD3 (1 μg/ml; BD Biosciences) and anti-CD28 (0.5 μg/ml; BD Biosciences) and differentiated into Th17 cells by adding recombinant human TGF-β (1 ng/ml), recombinant mouse (rm)IL-6 (20 ng/ml), anti–IFNγ (10 μg/ml) and anti–IL-4 (10 μg/ml) in T cell media composed of RPMI 1640 containing 10% FBS, 1% penicillin/streptomycin, 2 mM L-glutamine, 10 mM HEPES, 1 mM sodium pyruvate. Th1 cells were differentiated by adding rmIL-12 (10 ng/ml) and anti-IL-4 (10 μg/ml), Th2 cells were differentiated by adding rmIL-4 (10 ng/ml) and anti–IFNγ (10 μg/ml), and Th0 cells were in T cell media only at the time of activation. rmIL-23 was added at various concentrations (0–30 ng/ml) for Th17 cell differentiation. All antibodies and rmIL-23 was purchased from R and D systems. rmIL-6, and rhTGF-β were purchased from Peptrotech. In select experiments were 4,4′,4′′-(4-Propyl-[1*H*]-pyrazole-1,3,5-triyl)*tris*phenol (PPT) from Tocris was added, cells were activated as described above for 24 h. Cells were then cultured in phenol-red free RPMI containing 10% charcoal-stripped FBS, 1% penicillin/streptomycin, 2 mM L-glutamine, 10 mM HEPES, 1 mM sodium pyruvate under Th17 cell differentiating conditions with administration of PPT (30–100 pM) or vehicle (DMSO).

### Cytokine Measurements

Cytokine levels were measured from cell culture supernatants by ELISA using Duoset kits (R&D Systems). All ELISA experiments were performed according to the manufacturer's instructions. Any OD450 value less than the lower limit of detection was assigned half the value of the lowest detectable standard concentration on the standard curve.

### Flow Cytometric Analysis of IL-17A Production in Th17 Cells

Three days after Th17 cells differentiation, cells were restimulated with 50 ng/ml PMA, 1 μM ionomycin, and 0.07% of Golgi Stop for 4 h. Following restimulation, cells were stained with viability dye (Ghost Dye UV 450; Tonbo Biosciences), blocked with an anti-FcR Ab (clone 2.4G2), and surface stained with APC-Cy7 anti-CD3 (clone 145-2C11), FITC anti-CD4 (clone GK1.5), BV786 anti-CD90.2 (clone 53-2.1), and APC anti-IL-23R (clone 12B2B64). Cells were then fixed, permeabilized using the Foxp3/transcription factor staining kit (Tonbo Biosciences), and intracellularly stained PE-Cy7 anti–IL-17A (clone eBio17B7). Flow cytometry analysis was conducted on LSR II flow cytometer, and all data were processed using FlowJo software version 10. Antibodies were purchased from Thermo fisher (Invitrogen). In select experiments, Cell Tracer Violet dye was added per manufacturer's instructions (ThermoFisher) prior to Th17 cell differentiation. Th17 cell proliferation was determined by flow cytometry 3 days later by gating on viable, CD4+ IL-17A+ cells and measuring the proliferation index using FlowJo software.

### RNA Isolation and Real Time Quantitative PCR Analysis

Total RNA was isolated using a Trizol. cDNA was generated by using 100 ng of total RNA, and TaqMan quantitative PCR analysis of *Il23r, Cox20*, and *Gapdh* mRNA expression was conducted using commercially available primers and FAM/MGB probes (Applied Biosystems). Data were reported as relative expression normalized to the housekeeping gene *Gapdh*. In select experiments focused on *Let-7f* expression levels, miRNA was amplified per manufacturer's directions using the Quantabio qScript miRNA 2-step qPCR kit and commercially available primers and FAM/MGB probes (Applied Biosystems). Data were reported as relative expression normalized to the housekeeping gene *U6B*.

### Transfection of Let-7f Inhibitor or Cox20 siRNA Into CD4+ T Cells

Naïve CD4+ T cells were isolated from the spleens of the mice, FACS sorted and differentiated into Th17 cells. In select experiments, Th17 cells were transfected with 10 nM mirVana *Let-7f* inhibitor, 10 nM mirVana negative control, 1pmol Cox20 siRNA, or 1pmol non-targeting (NT) siRNA 24 h after Th17 cell activation and differentiation, using the Lipofectamine RNAiMAX Reagent. Cells were then harvested on day 3 for endpoints. Inhibitors and siRNA were purchased from ThermoFisher/Life Technologies and Lipofectamine RNAiMAX from Invitrogen.

### *In vivo* Administration of Hormone Pellets to Mice

Sixty day slow release pellets containing 17β-estradiol (0.1 mg) or vehicle pellets (Innovative Research Technologies) were surgically implanted subcutaneously into sham-operated, hormonally intact mice and gonadectomized female mice that lack ovaries and ovarian hormones (24). Three weeks (21 days) after pellet implantation, naïve CD4+ T cells were isolated from the spleens of the mice, FACS sorted and differentiated into Th17 cells. Three days after Th17 cell differentiation, RNA was isolated from cells and *Cox20* mRNA relative expression was assessed by qPCR analysis.

### Metabolic Assays

Naïve CD4+ T cells were activated and differentiated into Th17 cells as described in the above section. After 3 days, 100,000 cells per well were plated on Cell-Tak coated plates. OCR and extracellular acidification rate were measured on the Agilent Seahorse XF96 analyzer using the Seahorse XF Cell Mito Stress Test kit as previously described (26). OCR and ECAR were assessed under basal conditions and after consecutive injections with 1.0 μM oligomycin (ATP synthase inhibitor), 1.5 μM FCCP (oxidative phosphorylation uncoupler) and 0.5 μM Rot/AntA (respiration inhibitors). Control wells with media lacking cells were used for background measurements.

### Statistical Analysis

Data are represented as mean ± SEM where groups were compared by one-way ANOVA with Tukey *post-hoc* analysis. For experiments in **Figure 4**, a two-way ANOVA with Tukey *post-hoc* analysis was also conducted. For all analysis, p < 0.05 was considered significant.

## Results

### ERα Signaling Increased IL-17A Protein Expression in Th17 Cells

**Figure 1:**
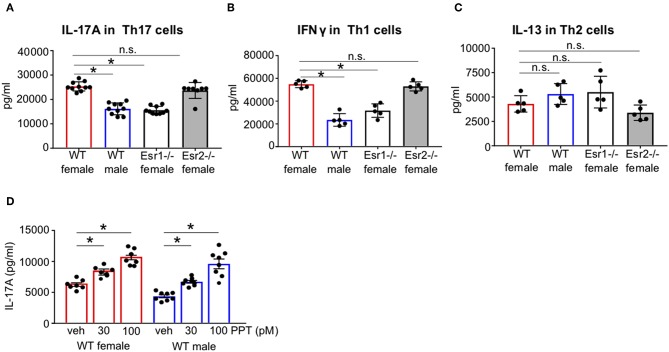
ERα signaling increased IL-17A protein expression in Th17 cells. FACS sorted naïve CD4+ CD62L+ T cells from WT female and male and *Esr1*^−/−^ and *Esr2*^−/−^ female mice were activated and differentiated into Th17, Th1, or Th2 cells for 3 days. **(A–C)** Cell culture supernatants were collected 3 days after differentiation and IL-17A, IFNγ, or IL-13 protein expression was measured by ELISA in Th17, Th1, and Th2 cells, respectively. **(D)** Twenty-four hours after Th17 cell activation and differentiation, PPT was administered to Th17 cells and culture supernatants were collected after 3 days for IL-17A analysis by ELISA. ^*^p < 0.05 ANOVA with Tukey *post-hoc* analysis. Data was pooled from two independent experiments. n.s. means not statistically significant.

There are conflicting findings on whether estrogen signaling enhances or attenuates Th17 cell differentiation and IL-17A production. Our previous study showed estrogen and progesterone increased IL-17A production (34–37). Therefore, we first determined if estrogen signaling through ERα and/or ERβ was important for IL-17A production from Th17 cells. CD4+ naïve T cells from WT female, WT male, *Esr1*^−/−^ female, and *Esr2*^−/−^ female C57BL/6J mice were differentiated into Th17 cells as previously described (24). IL-17A protein expression was significantly decreased in Th17 cells from WT male mice and *Esr1*^−/−^ female mice compared to WT female mice (Figure 1A). No differences in IL-17A protein expression were determined in Th17 from WT female and *Esr2*^−/−^ mice. In parallel, naïve T cells were also differentiated into Th1 and Th2 cells (24). IFNγ protein expression was also significantly decreased in Th1 cells from WT male and *Esr1*^−/−^ female mice compared to Th1 cells from WT female (Figure 1B). However, IL-13 protein expression was similar in Th2 cells from all groups (Figure 1C). Th17 cells are plastic and may shift toward other T cell subsets. Therefore, we determined if IFNγ and IL-13, cytokines produced predominantly by Th1 and Th2, respectively, were increased in Th17 cells from WT female, WT male, or *Esr1*^−/−^ female mice using ELISA. Th17 cells from all groups of mice had IFNγ and IL-13 values below the limit of detection (data not shown). These findings suggest that ERα, but not ERβ deficiency, decreased IL-17A production in Th17 cells and IFN-γ production in Th1 cells. However, deletion of ERα or ERβ signaling had no effect on IL-13 protein expression in Th2 cells. Next, we differentiated naïve T cells from WT male and female mice to become Th17 cells in the presence of the ER-α agonist PPT or vehicle (DMSO). IL-17A production was significantly increased in PPT treated Th17 cells from both WT female and WT male mice (Figure 1D), showing that ER-α signaling is important in increasing Th17 cell production of IL-17A.

### ERα Deficiency Decreased the IL-17A+ CD4+ T Cell Proliferation

**Figure 2:**
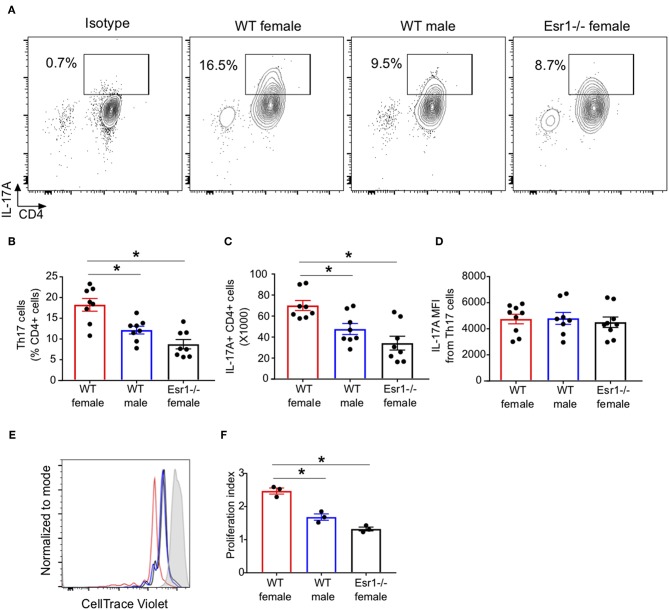
ERα deficiency decreased proliferation of Th17 cells. Th17 cells were differentiated for 3 days from WT female, WT male and *Esr1*^−/−^ female mice and then restimlulated with PMA, ionomycin, and golgi-stop. **(A)** Representative flow gating of IL-17A cytokine expression in viable CD3+CD4+ T cells (Th17 cells). **(B,C)** Frequency and total numbers Th17 cells. **(D)** IL-17A mean fluorescent intensity (MFI) in Th17 cells. ^*^p < 0.05 ANOVA with Tukey *post-hoc* analysis. Data was pooled from two independent experiments. n.s. means not statistically significant. **(E,F)** Th17 cell proliferation was measured using cell trace violet. **(E)** Representative histogram of cell trace violet staining on IL-17A+ CD4 cells 3 days after differentiation. **(F)** Proliferation index of Th17 cells. Data shown is from one of two experiments conducted. ^*^p < 0.05 ANOVA with Tukey *post-hoc* analysis.

ERα signaling increased CD4+ T cell proliferation and exacerbated colitis (34). Therefore, we next determined if ERα deficiency decreased the number of IL-17A+ CD4 cells (Th17 cells) or IL-17A production from each cells by conducting flow cytometry on Th17 cells restimulated with PMA, ionomycin, and golgi-stop for 4 h. Th17 cells from *Esr1*^−/−^ female mice had a significant decrease in the percentage and total number of IL-17A+ Th17 cells compared to WT female mice (Figures 2A–C). Th17 cells from *Esr1*^−/−^ female mice also had similar percentages and total numbers of IL-17A+ Th17 cells as WT male mice. However, the mean fluorescent intensity (MFI) was similar in Th17 cells from WT female, WT male, and *Esr1*^−/−^ female mice (Figure 2D). Since the number of IL-17A+ Th17 cells, but not MFI production, was decreased in Th17 cells from *Esr1*^−/−^ female mice compared to Th17 cells from WT female mice, we next determined cell proliferation of Th17 cells from WT female, WT male, and *Esr1*^−/−^ female mice. Cell proliferation, as measured by proliferation index, was significantly increased in IL-17A+ Th17 differentiated cells from WT female mice compared to male and *Esr1*^−/−^ female mice (Figures 2E,F), but not in IL-17A negative cells (data not shown). Combined these data showed that ERα deficiency decreased IL-17A production and limits cell proliferation of Th17 cells.

### ERα Deficiency Attenuated IL-23R Expression and IL-17A Protein Expression in Th17 Cells

**Figure 3:**
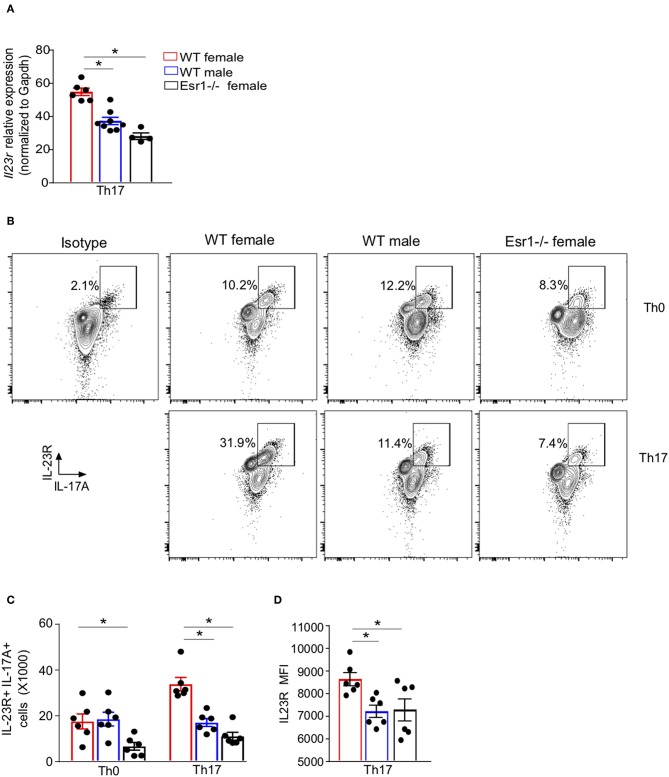
IL-23R surface expression was decreased on Th17 cells from *Esr1*^−/−^ mice. Naïve T cells were activated (Th0) and in select well-differentiated to become Th17 cells. **(A)** *Il23*r mRNA relative expression normalized to *Gapdh*. **(B)** Representative flow plots of IL-23R+ IL-17A+ CD3+ CD4+ viable T cells. **(C)** Total number of IL-23R+ IL-17A+ T cells. **(D)** MFI IL-23R in IL-23R+ IL-17A+ Th17 cells. ^*^p < 0.05 ANOVA with Tukey *post-hoc* analysis. Data was pooled from two independent experiments.

17β-estradiol and progesterone increased IL-17A protein in Th17 cells by upregulating IL-23R expression (24), but it remained unclear whether ERα signaling regulated IL-23R expression. We hypothesized that ERα signaling promotes IL-23R surface expression in Th17 cells, leading to increased IL-17A+ Th17 cells. To determine the effect of ERα signaling on *Il23r* mRNA relative expression in Th17 cells, we conducted qPCR on Th17 differentiated cells from WT female, WT male, and *Esr1*^−/−^ female mice. *Il23r* mRNA relative expression was significantly increased in Th17 cells from WT female mice compared to Th17 cells from WT male and *Esr1*^−/−^ female mice (Figure 3A). We confirmed these findings by measuring IL-23R surface expression by flow cytometry on viable Th0 and Th17 cells from WT female, WT male and *Esr1*^−/−^ female mice after 3 days of culture. IL-23R surface expression was also increased on Th17 cells compared to Th0 cells (Figures 3B,C). Further, the number of IL-23R+ Th17 cells as well as the IL-23R MFI was increased on Th17 cells from WT female mice compared to Th17 cells from WT male and *Esr1*^−/−^ female mice (Figures 3B–D). Combined these data showed ERα deficiency decreased the number of IL-23R+ Th17 cells.

**Figure 4:**
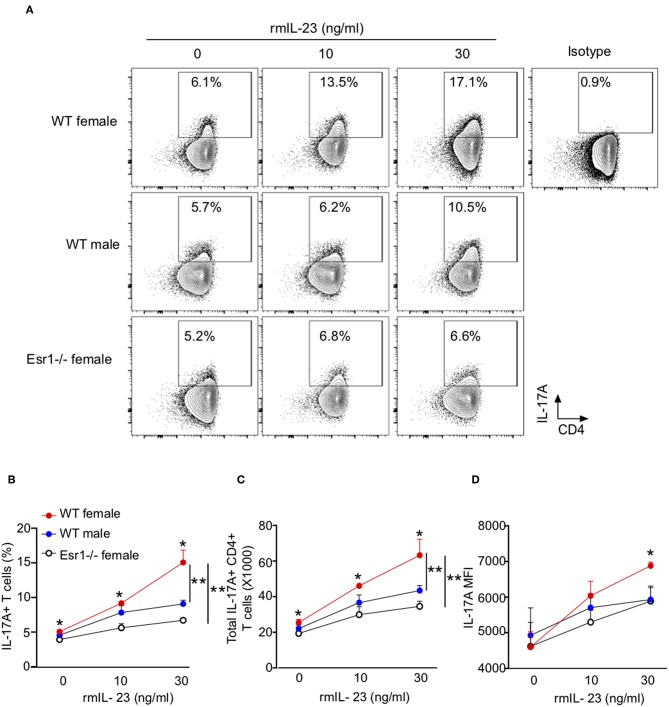
ERα deficiency attenuated IL-17A production in Th17 cells. Th17 cells were differentiated from WT female, WT male and *Esr1*^−/−^ female mice for 3 days in the presence of varying concentrations of rmIL-23 (0, 10, and 30 ng/ml). **(A)** Representative flow gating of IL-17A expression in viable Th17 cells. **(B,C)** Frequency and total numbers of IL-17A+ Th17 cells following rmIL-23 treatment. **(D)** MFI of IL-17A in Th17 cells. ^*^p < 0.05 ANOVA with Tukey *post-hoc* analysis. ^**^p < 0.05 two-way ANOVA. Data shown is one representative experiment of 3 independent experiments.

Since IL-23 is not required for Th17 cell differentiation, rather it results in increased IL-17A protein expression (38), we hypothesized that ERα signaling increased IL-23R expression leading to increased IL-17A production. To test this hypothesis, we measured IL-17A production from Th17 cells differentiated with rmIL-23 ranging from 0 to 30 ng/ml. At baseline (0 ng/ml), WT female mice had significantly increased IL-17A production compared to Th17 cells from WT male and *Esr1*^−/−^ female mice (Figures 4A–C). Further, higher concentrations of rmIL-23 significantly increased the frequency and total numbers of IL-17A+Th17 cells more in WT female compared to the Th17 cells from WT male and *Esr1*^−/−^ female mice (Figures 4A–C). The MFI of IL-17A staining was also significantly increased in WT female mice compared to WT male and *Esr1*^−/−^ female mice at 30 ng/ml rmIL-23 but not 10 ng/ml rmIL-23 (Figure 4D).

T While naïve CD4 T cells from WT female, WT male, and *Esr1*^−/−^ mice were all administered the same cocktail of cytokines and antibodies for Th17 cell differentiation, ~20% of the cells from WT female mice produced IL-17A and fewer cells in WT male and *Esr1*^−/−^. This level of IL-17A production is similar to what has been reported by others (26, 39), but the low percentage of IL-17A+ cells led us to determine if activated CD4+ T cells under Th17 differentiation conditions were converting into other T cell subsets, including Th1, Th2, and Treg cells (30, 40, 41). Therefore, we also determined if Th17 cells from WT female, WT male, and *Esr1*^−/−^ mice expressed T-bet, Gata3, RORγT, and Foxp3, transcription factors important for Th1, Th2, Th17, and T regulatory cells, respectively (42). Th17 cells from WT female, WT male, and *Esr1*^−/−^ female mice had a significant increase in RORγT expression levels in the presence or absence of IL-23 compared to Th0 (Supplemental Figure 1). However, there was no difference in RORγT expression in Th17 cells from WT female, WT male, or *Esr1*^−/−^ female mice. Low levels of Foxp3 single positive and Foxp3/RORγT double positive cells were detected in Th17 cells from WT female, WT male, and *Esr1*^−/−^ mice, but no differences were determined between groups. Upregulation of Gata3 and T-bet were not observed in Th17 cells from WT female, WT male, and *Esr1*^−/−^ female mice (data not shown). Combined, these data show that ERα deficiency decreased IL-17A production.

### ERα Deficiency Increased *Let-7f* miRNA Expression and Inhibition of *Let-7f* Increased IL-17A Production

**Figure 5:**
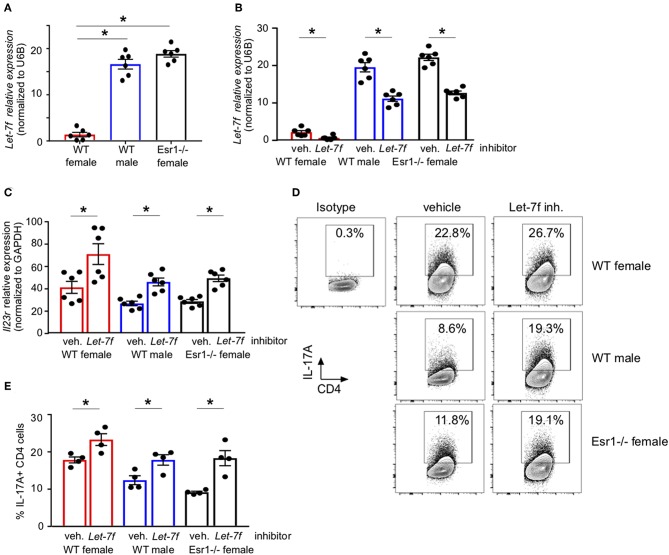
ERα deficiency increased *Let-7f* miRNA expression and inhibition of *Let-7f* increased IL-17A production in Th17 cells. **(A)** *Let-7f* miRNA relative expression in Th17 cells normalized to *U6B* miRNA expression. **(B–E)** Th17 cells were differentiated from WT female, WT male and *Esr1*^−/−^ female mice for 3 days in the presence of Let-7f inhibitor or vehicle control. **(B)** *Let-7f* miRNA relative expression in Th17 cells normalized to *U6B* miRNA expression. **(C)** *Il23r* relative expression in Th17 cells normalized to *Gapdh*. **(D-E)** IL-17A+ CD4 T cells dots plots and quantification. ^*^p < 0.05 ANOVA with Tukey *post-hoc* analysis. Data was pooled from two independent experiments.

We have previously published that *Let-7f*, a microRNA that has been shown to regulate IL-23R expression (24), was significantly increased in Th17 cells from gonadectomized female mice administered 17β-estradiol and progesterone hormone pellets compared to Th17 cells from gonadectomized female mice administered vehicle pellets. Estrogen signaling inhibited miRNAs, including *Let-7f* (43), and Let-7f negatively regulated IL-23R (31). Therefore, we measured *Let-7f* miRNA expression in differentiated Th17 cells from WT female, WT male and *Esr1*^−/−^ female mice and found *Let-7f* miRNA expression was significantly decreased in WT female mice compared to WT male and *Esr1*^−/−^ female mice (Figure 5A). Based on these findings, we hypothesized that ERα signaling inhibits *Let-7f* expression to promote IL-23R and IL-17A expression in Th17 cells. To test this hypothesis, we transfected either a *Let-7f* miRNA inhibitor or control inhibitor miRNA into Th17 differentiated cells and determined IL-23R expression and IL-17A production from Th17 cells. *Let-7f* miRNA inhibitor significantly decreased *Let-7f* expression (by ~50%) in Th17 cells from WT female, WT male, and *Esr1*^−/−^ female mice (Figure 5B). With Let-7f inhibition, *Il23r* mRNA expression and IL-17A+ T cells were significantly increased in Th17 cells from WT female, WT male, and *Esr1*^−/−^ female mice (Figures 5C–E). These data suggest that ERα signaling negatively regulated *Let-7f* expression, leading to increased Th17 cells.

### ERα Deficiency Decreased Mitochondrial Respiration in Th17 Cells

**Figure 6:**
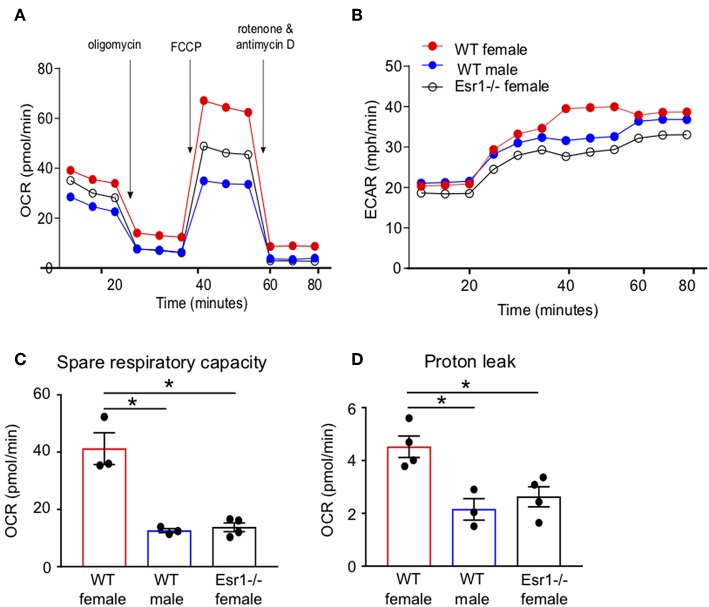
Mitochondrial respiration and proton leakage were decreased in Th17 cells from *Esr1*^−/−^ mice. Th17 cells were differentiated from WT female, WT male and *Esr1*^−/−^ for 3 days and OCR was determined by Seahorse extracellular flux analysis. Representative plot of OCR **(A)** and ECAR **(B)** over time in Th17 cells with addition of oligomycin (1 μM), FCCP (1.5 μM) and Rot/AntA (0.5 μM). **(C,D)** Quantification of spare respiratory capacity and proton leak in Th17 cells. Data are taken from one representative experiment of the three independent experiments conducted. ^*^p < 0.05, ANOVA with Tukey *post-hoc* analysis.

We next wanted to determine if ERα signaling increased IL-17A production and/or Th17 cell differentiation through mechanisms other than IL-23R. Transcriptomics analyses previously showed ERα signaling increased expression of genes associated with TCR signaling, including *Cd3e, Cd4, Zap70*, and *Cd69*, in Th17 cells (34), suggesting a T-cell intrinsic role for ERα signaling increasing T cell activation. Mitochondrial respiration, including glycolysis and glutaminolysis, is increased upon effector T cell activation (25, 26, 44). Based on the previous findings and data, we hypothesized that ERα signaling increased mitochondrial respiration leading to increased Th17 cell differentiation and IL-17A production. To test this hypothesis, we measured the oxygen consumption rate (OCR), a measure of mitochondrial respiration, in naïve T cells being differentiated into Th17 cells from WT female, WT male and *Esr1*^−/−^ female mice. The OCR curve was higher in T cells from WT female mice compared to WT male mice and *Esr1*^−/−^ female mice (Figures 6A,B). Extracellular acidification rate (45), a measure of glycolysis, was similar in Th17 differentiating cells from WT female, WT male, and *Esr1*^−/−^ female mice (Figure 6B). The spare respiratory capacity and proton leakage were also significantly increased in Th17 differentiating cells from WT female mice compared to cells from WT male and *Esr1*^−/−^ female mice (Figures 6C,D). Combined, the results suggested that ERα signaling increased mitochondrial respiration in Th17 differentiating cells and provided a mechanism for increased IL-17A production.

### Cox20 Is Decreased in *Esr1^−/−^* Mice and Regulates IL-17A Production From Th17 Cells

**Figure 7:**
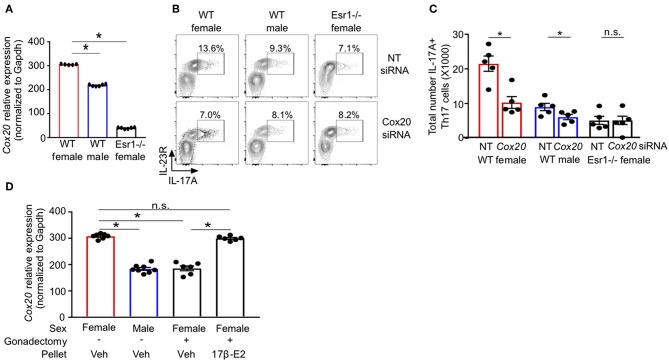
ERα deficiency decreased Cox20 expression, leading to decreased IL-17A production from Th17 cells. **(A)** *Cox20* mRNA relative expression normalized to *Gapdh* in Th17 cells. **(B,C)** Th17 cells were differentiated from WT female, WT male and *Esr1*^−/−^ female mice for 3 days in the presence of Cox20 or non-targeting (NT) siRNA and IL-23R+ IL-17A+ Th17 cells were determined by flow cytometry. **(D)** *Cox20* mRNA expression from Th17 differentiated cells from WT female and male mice that had undergone sham or gonadectomies and been administered slow-release vehicle or 17β-estradiol pellets. Data pooled from 2 independent experiments. ^*^p < 0.05, ANOVA with Tukey *post-hoc* analysis. n.s. means not statistically significant.

RNA-sequencing of Th17 cells from healthy individuals revealed that *COX20*, a chaperone protein involved in formation of complex IV (cytochrome c oxidase) the terminal complex that pumps protons and controls mitochondrial respiration (46–48), was significantly increased in Th17 cells from women compared to Th17 cells from men (Supplemental Figure 2). These data suggested a sex difference in mitochondrial function, and therefore we measured *Cox20* mRNA relative expression in Th17 cells from WT female, WT male and *Esr1*^−/−^ female mice. *Cox20* expression was significantly increased in Th17 cells from WT female mice compared to Th17 cells from WT male mice and *Esr1*^−/−^ female mice (Figure 7A). Based on these results, we hypothesized that inhibition of Cox20 would decrease IL-17A production to a greater degree in Th17 cells from WT female mice compared to Th17 cells from WT male and *Esr1*^−/−^ female mice. To test this hypothesis, naïve T cells from WT female, WT male, and *Esr1*^−/−^ female mice were activated and differentiated to become Th17 cells. One day later, cells were transfected with 1pmol Cox20 siRNA or NT siRNA and cells continued to differentiate for 2 additional days. Cox20 inhibition was determined to be 55 ± 9% by measuring *Cox20* expression via qPCR in T cells transfected with Cox20 and NT siRNA. CD4+ T cells underwent the same protocol with Cox20 siRNA transfection, and 2 days later cells were restimulated with PMA and ionomycin in the presence of golgi-stop for 4 h and IL-17A production from IL-23R+ Th17 cells was measured by flow cytometry. Cox20 inhibition significantly decreased IL-17A+ Th17 cells in female mice (Figures 7B,C). While there was a trend for Cox20 decreasing IL-17A+ IL-23R+ Th17 cells from WT male and *Esr1*^−/−^ female mice, there was no significant difference.

We next determined if estrogen increased *Cox20* expression in Th17 cells; therefore, we administered slow-release 17β-estradiol or vehicle hormone pellets to sham-operated female and male mice and gonadectomized female mice for 21 days. Naïve T cells were then differentiated into Th17 cells, and *Cox20* mRNA expression was measured. *Cox20* expression was significantly increased in Th17 cells from sham-operated female mice administered vehicle hormone pellets and gonadectomized female mice administered 17β-estradiol compared to Th17 cells from sham-operated male mice and gonadectomized female mice administered vehicle pellets (Figure 7D). These data showed that 17β-estradiol and ER-α signaling increased Cox20 expression in Th17 cells.

## Discussion

A female predominance exists in autoimmune diseases and severe phenotypes of asthma, with increased Th17 cells and IL-17A production (1–5). Estrogen signaling enhances effector T cell responses and Th17 cell differentiation (24, 34), and we expand upon these findings to show that ERα deficiency decreased IL-17A production and Th17 cell differentiation by decreasing IL-23R expression on Th17 cells as well as decreasing mitochondrial respiration and Th17 cell proliferation. These results provide additional mechanisms to explain how ERα signaling enhances Th17 cell differentiation and IL-17A production, leading to increased Th17 cell-mediated disease pathogenesis.

The literature has discordant findings on the role of estrogen signaling on IL-17A production and Th17-mediated diseases. Our findings showed that Th17 cells from *Esr1*^−/−^ female mice had no change in RORγT expression compared to Th17 cells from WT female and WT male mice. However, a previous study showed that estrogen inhibited Th17 cell differentiation by complexing with a repressor of estrogen receptor element and binding to estrogen response elements on the *Rorc* promoter region to attenuate *Rorc* transcription (49). It is unclear why these differences are seen, but variations in mouse strain, T cell culturing conditions, and concentrations of estrogen likely contribute to the observed differences.

The role of estrogen in autoimmune and allergic diseases also varies *in vivo*. In mouse models of multiple sclerosis, EAE, ERα signaling suppressed EAE development by increasing PD-1 signaling on cognate CD4+ (Foxp3-) T cells, and that inhibition of PD-1 signaling removed the estrogen dependent inhibition of EAE (50). ERβ expression in CD4+ T cells was required for DHEA, a hormone upstream of testosterone and estrogen,-mediated suppression of Th17 cells and EAE (51), and estrogen suppressed IL-17-mediated osteoclast differentiation which is important in osteoporosis (52). However, other studies showed that ERα signaling in CD4+ T cells inhibited Th17 or Th1 mediated inflammation in mouse model of colitis (34), but ovarian hormones (including estrogen) had no direct effect on house-dust mite induced Th2-mediated airway inflammation *in vivo* (53). Our findings were consistent with these studies as deficiency of ERα, but not ERβ, signaling decreased IL-17A and IFNγ protein expression from Th17 and Th1 cells, respectively, but had no effect on IL-13 protein expression from Th2 cells. Further, Th17 cells from *Esr1*^−/−^ female mice were not transitioning toward Th1 or Th2 cells, since no IFNγ, T-bet, IL-13, or GATA3 was detected. Rather, proliferation of Th17 cells in *Esr1*^−/−^ female mice was decreased compared to Th17 cells from WT female mice.

ERα signaling also promoted IL-23R surface expression in Th17 cells. *Let-7f* negatively regulates IL-23R surface expression and IL-17A production from Th17 cells, and *Let-7f* expression is decreased by estrogen and progesterone (24, 31, 54). Th17 cells from *Esr1*^−/−^ female mice had increased *Let-7f* miRNA expression compared to WT female mice. Further, inhibition of *Let-7f* increased *Il23r* mRNA expression and IL-17A producing Th17 cells from WT female, WT male, and *Esr1*^−/−^ female mice, demonstrating ERα that signaling regulated IL-17A production by decreasing *Let-7f*.

Our findings also showed that even in the absence of rmIL-23, IL-17A protein expression was increased in Th17 cells from WT female mice compared to WT male and *Esr1*^−/−^ female mice. Th17 cells are differentiated in the presence of IL-6 and TGF-β, while TGF-β3 and IL-23 promote the generation of pathogenic Th17 cells by stabilizing the Th17 phenotype and limiting T cell plasticity (38, 55). Transcriptional profiling of Th17 cells differentiated with TGF-β and IL-6 in the presence or absence of IL-23 further showed that IL-23 promoted the expression of serum glucocorticoid kinase 1 (SGK1), a serine/threonine kinase found that is highly expressed in IL-23R+IL-17A+ Th17 cells and γδ T cells, leading to increased memory Th17 effector responses (56). These findings are in alignment with previous studies where IL-23R was shown to be important for optimal IL-17A production and maintenance of the Th17 cells phenotype but not for Th17 cell differentiation (38, 55), and suggested IL-23 independent mechanisms by which ERα signaling promotes IL17A production in Th17 cells.

Activated T cells undergo metabolic reprogramming during Th17 cell differentiation, including glycolysis, glutaminolysis, oxidative phosphorylation, and *de novo* fatty acid synthesis (25–30, 44). ERα signaling has recently been shown to regulate mitochondrial activity in CD4+ T cells where TCR-mediated activation of naïve CD4+ T cells from ERα^fl/fl^ or CD4-creERα^fl/fl^ significantly increased mitochondrial respiration in ERα^fl/fl^ compared to CD4-creERα^fl/fl^ (34). In this report, we expand these findings and show that ERα deficiency decreased mitochondrial respiration and proton leakage in Th17 differentiating cells. Our data also determined that Th17 cells were not expressing Foxp3 in WT male or *Esr1*^−/−^ female mice, and therefore it seems unlikely that estrogen signaling regulates *de novo* fatty acid synthesis in Th17 cells (30). Additional experiments to confirm this need to be conducted. We also showed that estrogen signaling increased expression of *Cox20* in Th17 cells and that the inhibition of Cox20 further decreased IL-17A production in IL-23R+ Th17 cells from WT females, providing potential targets that can specifically be inhibited in Th17 cells may allow for Th17-cell specific inhibition in Th17-dominant diseases.

Various epigenetic mechanisms are associated with T cell differentiation and plasticity, including methylation, of DNA and histones (57, 58). Our results showed that *Esr1*^−/−^ Th17 cells were not becoming Th1, Th2, or Treg cells; however, ERα signaling likely regulates other mechanisms beyond IL-23R signaling and Th17 cell proliferation and mitochondrial respiration. Histone modifications specifically are well-implicated in regulating T cell differentiation and previous studies in effector T cells from mice showed that H3K4me3 marks were significantly increased at *Rorc* and *Il17a* in Th17 cells. In contrast, H3K27me3 marks were significantly increased in the promoter regions for genes that are important for Th1 cells (*Ifn*γ and *Tbx21)* and Th2 cells (*Il4* and *Gata3)* (57). Therefore, additional studies are needed to determine if ERα signaling regulates histone modifications important for T cell differentiation. ERα signaling increases IL-17A protein expression in Th17 cells by enhancing IL-23R expression and promoting mitochondrial respiration. Our results align with epidemiological and clinical findings in patients with asthma showing increased prevalence of severe asthma in females after puberty, pre-menstrual worsening of symptoms, increased asthma control with oral contraceptive use, and fluctuations of asthma control in perimenopausal women (5–12). Further, these findings provide personalized approaches to treating patients with Th17-mediated diseases, particularly women, during different reproductive phases of life.

## Data Availability Statement

The raw data supporting the conclusions of this manuscript will be made available by the authors, without undue reservation, to any qualified researcher.

## Ethics Statement

The studies involving human participants were reviewed and approved by Vanderbilt University Institutional Review Board Policies. The patients/participants provided their written informed consent to participate in this study. The animal study was reviewed and approved by The International Animal Care and Use Committee at Vanderbilt University Medical Center.

## Author Contributions

HF and DN created the concept of the paper. HF conducted the experiments, literature research, and wrote the manuscript. J-YC and JD assisted with experiments and mouse genotyping. PW assisted with analysis of RNA Sequencing data. DC and JR provided supplies, assistance, and expertise with metabolic assays. VG, JR, and DN revised the manuscript.

### Conflict of Interest

The authors declare that the research was conducted in the absence of any commercial or financial relationships that could be construed as a potential conflict of interest.

## Abbreviations

ATP: adenosine triphosphate
ERα: estrogen receptor α
ERβ: estrogen receptor β
FCCP: carbonyl cyanide 4-(trifluoromethoxy) phenylhydrazone
FBS: Fetal bovine serum
MFI: mean fluorescence intensity
NT: non-targeting
OCR: oxygen consumption rate
PBS: phosphate-buffered saline
PMA: phorbol 12-mystriate 13-acetate
P4: Progesterone
qRT-PCR: quantitative real-time PCR
rm: recombinant mouse
Th1: T helper 1
Th2: T helper 2
Th17: T helper 17
Veh: vehicle
WT: wildtype
n.s.: not significant.
