# Supplementary material for: ERα Signaling Increased IL-17A Production in Th17 Cells by Upregulating IL-23R Expression, Mitochondrial Respiration, and Proliferation
Source: Front Immunol. 2019 Nov 27;10:2740. doi: 10.3389/fimmu.2019.02740 (PMC6892971; doi:10.3389/fimmu.2019.02740)
Supplement: Supplementary file 1 [file Data_Sheet_1.docx]

***Supplemental Materials***

**ERα signaling increased IL-17A production in Th17 cells by upregulating IL-23R expression and promoting mitochondrial respiration and proliferation**

Hubaida Fuseini^1^, Jacqueline-Yvonne Cephus^2^, Pingsheng Wu^2^, J. Brooke Davis^2^, Diana, C. Contreras^1^, Vivek D. Gandhi^2^, Jeffrey C. Rathmell^1^, Dawn C. Newcomb^1,2*^

^1^Department Pathology, Microbiology and Immunology, Vanderbilt University, Medical Center North, 1161 21^st^ Avenue MCN B-1222, Nashville, TN 37232

^2^Department of Medicine, Vanderbilt University Medical Center, Medical Center North, 1161 21^st^ Avenue B-1222, Nashville TN, 37232

***Correspondence:**

Dr. Dawn C. Newcomb

dawn.newcomb@vumc.org

Keywords: Th17, estrogen, estrogen receptor alpha, IL-23R, IL-17A, oxygen consumption rate, COX20, cytochrome c oxidase

**1 Supplemental Methods**

**Flow Cytometric Analysis of IL-17A Production in Th17 cells**

Three days after Th17 cells differentiation, cells were harvested and stained with viability dye (Ghost Dye UV 450; Tonbo Biosciences), blocked with an anti-FcR Ab (clone 2.4G2), and surface stained with FITC anti-CD4 and APC anti-IL-23R. Cells were then fixed, permeabilized using the Foxp3/transcription factor staining kit (Tonbo Biosciences), and intracellularly stained PE-Cy7 anti–Foxp3, PE anti-RORγT, BV421 anti-T-bet, and PE-CF594 anti-GATA3 antibodies. Flow cytometry analysis was conducted on LSR II flow cytometer, and all data were processed using FlowJo software version 10.

**Recruitment of participants**

Healthy participants (aged 18-45 years) were recruited at Vanderbilt University Medical Center. Participants were excluded for viral and bacterial symptoms in the previous week or any Th17-associated disease, including multiple sclerosis, psoriasis, systemic lupus erythematosus or rheumatoid arthritis. Women were excluded if pregnant, breast-feeding, taking hormonal birth control medications, on estrogen replacement therapy, menopausal or if they had undergone an oophorectomy or hysterectomy. All participants were consented in accordance with Vanderbilt University Institutional Review Board Policies.

**Human Th17 cell differentiation and RNA isolation**

Human PBMCs were isolated from peripheral blood using Ficoll-Plaque Plus. Naïve CD4+ CD62L+ T cells were isolated from PBMCs by using a commercially available naïve T cell isolation kit (Miltenyi). Naïve T cells were activated using an activation/expansion kit (Miltenyi) with anti-CD3 and anti-CD28 bound to bead particles at a ratio of 1 bead particle per 2 cells. CD4+ T cells were cultured and differentiated into Th17 cells with hIL-2 (10 ng/ml), rhIL-1β (10 ng/ml), rhTGF-β (1 ng/ml), rhIL-6 (10 ng/mL), rhIL-23 (10 ng/ml), anti-IFNγ (10 μg/ml) and anti-IL-4 (10 μg/ml) in complete RMPI 1640 T cell media containing 10% FBS, 1% penicillin/streptomycin, 2 mmol/L- L-glutamine, 10 mM HEPES, 0.1 mM nonessential amino acids and 1 mM sodium pyruvate. All antibodies and rhIL-1β, rhIL-6, and rhIL-23 were purchased from R & D systems. rhIL-2 and rhTGF-β were purchased from Peptrotech (Rocky Hill, NJ).

Total RNA was isolated using Trizol and RNA sequencing was conducted at Vanderbilt Technologies for Advanced Genomics Core. RNA sequencing (RNA-seq) libraries were prepared using 50 ng of total RNA and Illumina TruSeq Stranded mRNA Kit (Illumina) per manufacturer’s instructions, with mRNA enriched via poly(A) selection using oligo(DT) beads. RNA was then converted to cDNA, adenylated for adapter ligation, and PCR amplified. The libraries were sequenced using the HiSeq 2500 with 75 bp end reads with a depth of ∼30 million reads per sample. Real-Time Analysis (version 2.7.6; Illumina) was used for base calling, and analysis was completed using MultiQC v1.2. On paired FASTQ files, transcript abundance was estimated, and these read counts were imported into edgeR for differential expression analysis and normalized based on negative binomial distribution. The differential expression of genes between Th17 cells from women and men samples was assessed by estimating an exact test p value.

**2 Supplemental Figures**


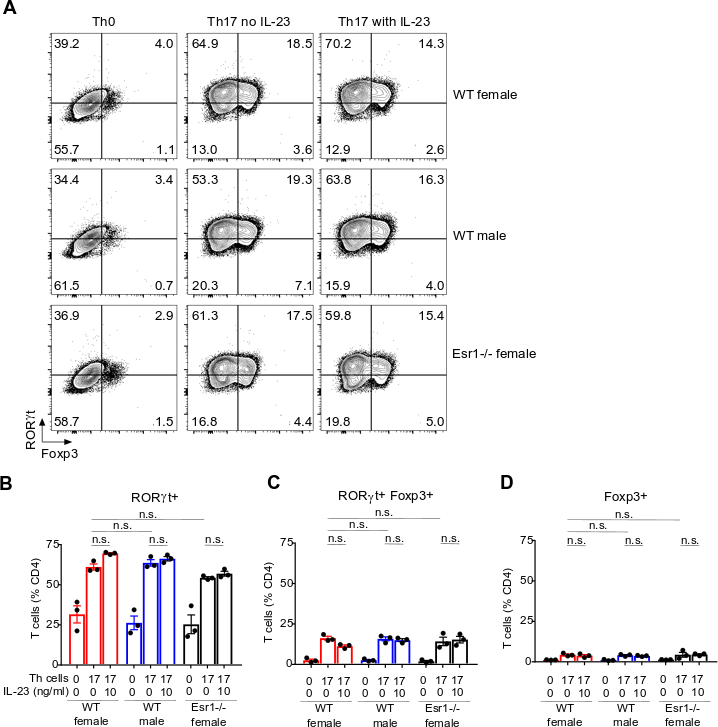


**Supplemental Figure 1: Analysis of transcription factor in Th17 differentiated cells from WT female, WT male, and Esr1-/- female mice.** Th0 and Th17 cells were differentiated in the presence of varying concentrations of rmIL-23 (0, 10 and 30 ng/ml). **(A)** Representative dot plots were pre-gated on CD4+ IL-23R+ cells. **(B-D)** Frequency of RORγt+, RORγt+Foxp3+, and Foxp3+ cells. *p<0.05 ANOVA with Tukey post hoc analysis. n.s. means not statistically significant.


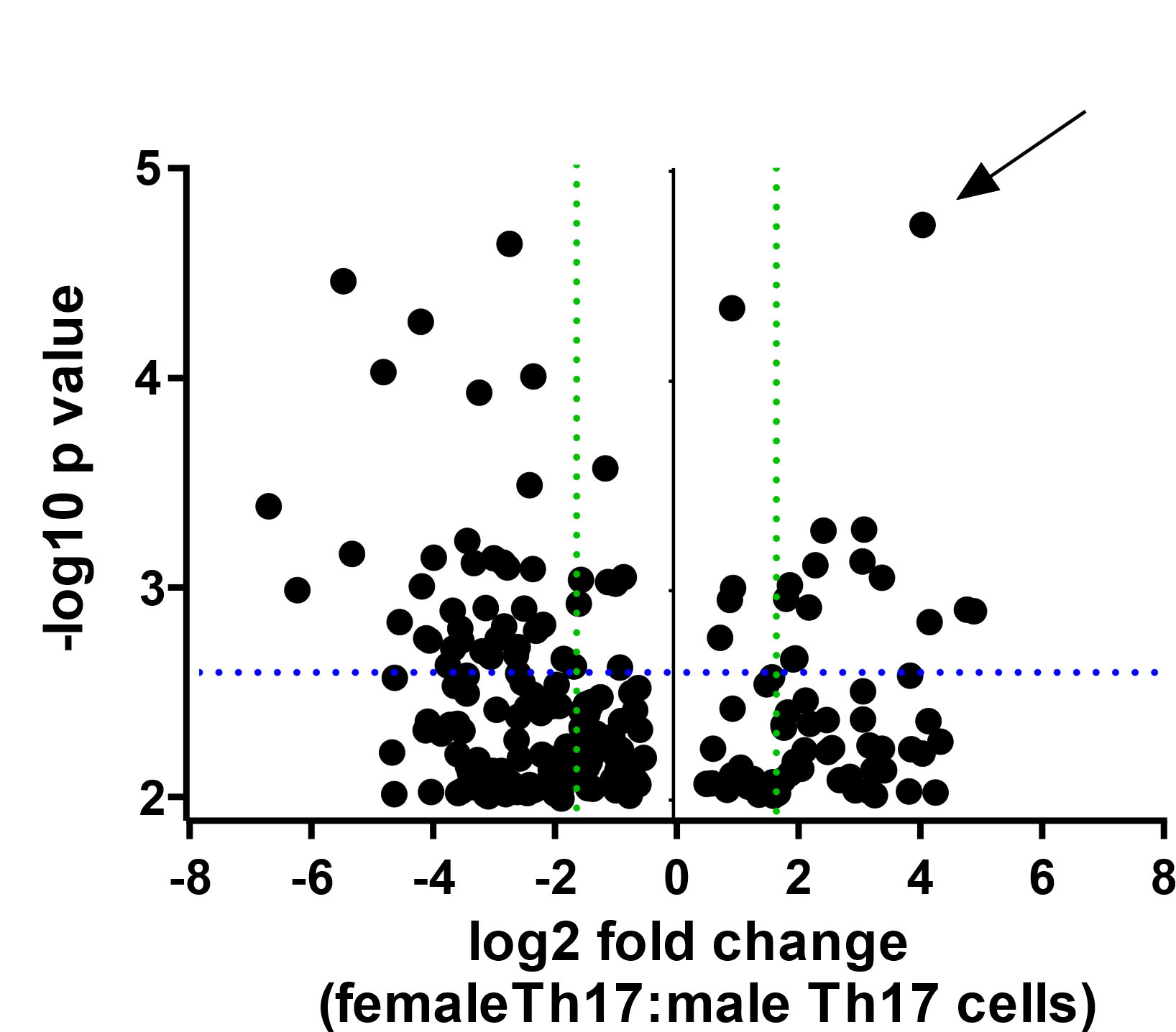


**Supplemental Figure 2:** Plot of differential expressed genes from cultured Th17 cells from healthy women compared to Th17 cells from healthy men. Y-axis shows -log_10_ p value with dotted blue line denoting p<0.05 and green dotted line denoting log2 fold change >2.
